# Understanding the funding characteristics of research impact: A proof-of-concept study linking REF 2014 impact case studies with Researchfish grant agreements

**DOI:** 10.12688/f1000research.74374.3

**Published:** 2022-09-20

**Authors:** Gavin Reddick, Dmitry Malkov, Beverley Sherbon, Jonathan Grant

**Affiliations:** 1 Interfolio UK, Cambridge, UK; 2 Science Policy Research Unit, University of Sussex Business School, Brighton, UK; 3 Different Angles, Cambridge, UK; 4 The Bennett Institute for Public Policy, University of Cambridge, Cambridge, UK

**Keywords:** Researchfish, REF, digital-data, linkage, impact, research characteristics

## Abstract

Background: All parts of the research community have an interest in understanding research impact whether that is around the pathways to impact, processes around impact, methods for measurement, describing impact and so on. This proof of concept study explored the relationship between research funding and research impact using the case studies submitted to the UK Research Excellence Framework (REF) exercise in 2014 as a proxy for impact.

Methods: The paper describes an approach to link the REF impact case studies with the underpinning research grants present in the Researchfish dataset, primarily using the publications captured in both datasets. Where possible the methodology utilised unique identifiers such as Digital Object Identifiers and PubMed ID’s, and where this was not possible the funding information within each publication was used.

Results: Through this automated approach 21% of the non-redacted case studies could be linked to a specific research grant. Additional qualitative analysis was then done for unlinked REF impact case studies, which involved reading the document to identify additional information to make the linkage. This approach was taken on 100 REF impact case studies selected at random and resulted in only seven having no identifiable research grants funding associated. The linked research grants were analysed to identify characteristics that are more frequently associated with these grants, than non-linked ones.

Conclusions: This analysis did point to some interesting observations such as the grant funding linked to REF impact case studies are more likely to be longer, higher financial value, have more publications and be more collaborative (amongst other characteristics). These findings should be used with caution at present and not be over interpreted, this is due to the sample size for this proof of concept study and some potential limitations on the data which were not addressed at this stage.

## Introduction

The purpose of this proof-of-concept study was to explore the relationship between research funding and research impact by linking Research Excellence Framework (REF) 2014 impact cases studies (ICS) with Researchfish Grant Agreements (GA). As such it builds on a long history of studies investigating factors associated with research impact ( Marjanovic *et al.,* 2009). For example, in the 1960s and 1980s there were a series of studies that examined the contributions research makes to society and what were the characteristics of that research. Some studies looked at the genesis of individual innovations ( Jewkes *et al*., 1958; Sherwin and Isenson, 1967; Illinois Institute of Technology, 1968; Comroe and Dripps, 1976; Battelle Laboratories, 1973), whilst others focused on better understanding the process through which research contributes to innovation i.e. research translation pathways and variables ( Evered *et al*., 1987; Narin, 1989; Arundel *et al*., 1995). In the 1990s and 2000s, the theme of measuring research impact – both quantitatively through economic analysis and qualitatively through case studies – began to dominate the scholarly literature (e.g. Mansfield, 1991; Herbertz and Müller-Hill, 1995; Buxton and Hanney, 1996; Grant *et al.,* 2000; Grant and Buxton, 2018; Hanney *et al.,* 2003a, 2003b; Wooding *et al*., 2004). By the 2010s some of these approaches began to be operationalised into national assessment through, for example, the introduction of impact into the UK’s REF and to a lesser extent the Australian Engagement and Impact Assessment ( Williams and Grant, 2018).

Bozeman *et al.*(1999) explained how these studies had moved through four incremental phases: 1) *historical descriptions* – tracing innovations back to their fundamental supporting inventions; 2) ‘ *research event’ based case-studies* – building a family tree of research events that led to an innovation; 3) *matched comparisons* – taking matched successful and unsuccessful innovations, tracing and comparing their development; and 4) *conventional case studies* – using action research, interviews, surveys, narrative descriptions – complemented with economic and bibliometric techniques in an attempt to increase methodological rigour and objectivity ( Grant and Wooding, 2010). Today we can perhaps add a fifth phase that is associated data linkage and datamining, facilitated by access to digital data ( King’s College London and Digital Science, 2015; Onken *et al.,* 2020). One of the best proponents of this is a recent study by Onken *et al.*(2020) that traced the long-term impact of research funded by the National Institute of General Medical Science by linking grant data with primary publications and associated citations (over a number of generations) with patents, and drug products approved by the US Food and Drug Administration.

Building on the opportunity presented by digital data, in the proof-of-concept study reported here we examined whether it was possible to link REF 2014 ICS with Researchfish GA, and where that occurs what are the characteristics of linked GA versus non linked GAs. We were motivated to undertake the study with an eye was kept on the impending outcomes of REF2021 and the anticipated publication of a further set of *circa* 7000 case studies. As described below, the first iteration of the study resulted in relatively low levels of linkage so it was not known whether the ‘unlinked’ case studies were ‘real’ i.e. do not have underpinning research grants associated with them or were an ‘artefact’, either of (i) the process used by the authors or (ii) have associated underpinning research grants that are not indexed on Researchfish. To test this a random sample of 100 ICS were selected to see whether they could be linked to GA though more in depth quantitative and qualitative approaches, that is either through semi-automated process or by hand. Based on this in-depth assessment a detailed comparison of the GAs that were linked to REF ICS vs all GAs in the Researchfish database was undertaken. This elucidated a number of interesting observations about the characteristics between research funding and research impact, although it must be stressed that these observations need to be validated and thus should be treated with caution.

### Data sources

The two key data sources for this study were the REF 2014 ICS and Researchfish GA. The REF reviews the research quality of UK universities every 5-6 years. It matters not only as a signal of the reputation of an institution, but also because it determines the allocation of government block grant funding to universities, known as ‘QR funding’ (quality related research funding). The REF has been running in various iterations since 1986, but critically in the 2014 exercise (and the current 2021 iteration) the assessment of societal impact was included. REF is organised around four main panels (A to D) representing broad cognate disciplines (such as Arts and Humanities, Panel D) and 36 units of assessment (UOA, or sub panels) for specific disciplines (such as History, UOA 30; REF2014).

Impact was assessed through 6,975 ICS that were 4-5 page summaries of the contribution research had made on society over a 20 year period ( King’s College London and Digital Science, 2015). The ICS are published through the online REF2014 database, which includes an API allowing for data extraction, linkage and analysis. The database only contains ICS that were not redacted and where the submitting university had given permission for them to be published, resulting in 6637 ICS that could be analysed for the purpose of this study. One section of the ICS was the ‘underpinning research’ which typically contained citations to publications in the (peer reviewed) literature, which included where available digital object identifiers (DOI) which could facilitate data linkage.

Researchfish is an online platform designed to enable researchers to report the outcomes of their work across multiple funders, to re-use their data for their own purposes and to have control over who sees and accesses the data. Researchfish is essentially a data collection tool and supporting service for organisations to track research and evidence impact. Research outputs (and outcomes and impact) are gathered through a standard ‘question set’ initially developed by funding institutions through a consultative process, within subsequent ongoing governance from the Researchfish Question Set Subgroup which is comprised of stakeholders from funders and research organisations that use the system. This question set has 16 main outcome types e.g. publications, collaborations, IP, engagement activities and so on with each being broken down into sub-types, of which there are 103 in total. A researcher, or one of their delegates, can add, edit and delete entries, and crucially, attribute entries to research grants and awards (GAs). This collation and attribution of research outputs and outcomes serves a number of purposes. Research funders can capture a range of data that have been submitted by the researchers they fund – from publications, policy impact to products and interventions – enabling them to evaluate the impact of their research funding by various units of assessment (e.g., disciplinary focus, research funding mechanism, host institution etc). Research publications are automatically populated using web scraping technologies and the researcher or delegate confirms whether the publication is associated with the research grant. Where that automation occurs the DOI is also captured thus facilitating linking with other external datasets including potentially the REF 2014 ICS.

Currently Researchfish has data on over 195,000 Grant Agreements, with over 80% of them from the UK. These UK data report on 268,000 different outputs, outcomes or impact before December 31 ^st^ 2013 (the cut off period for REF 2014). All the major funders in the UK (i.e. UKRI, Wellcome Trust and other medical research charities) use Researchfish and over the period 2006-2013 this accounted for between £2.5-£4.0 billion of research funding each year. It, should, however be noted that Researchfish does not cover research that is funded by other means, for example block grants to universities (QR funding), direct donation from philanthropists and other self initiated research.

## Methods

**Figure 1.**
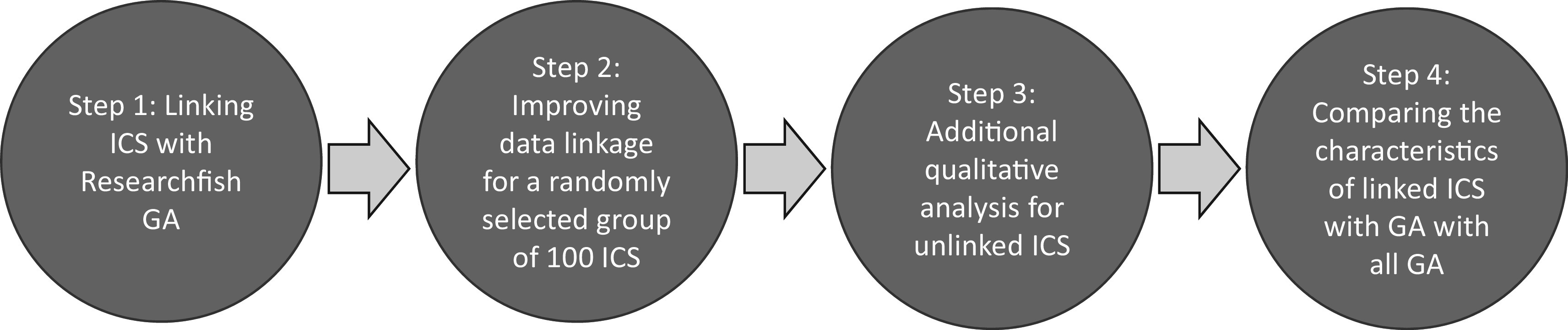
Schematic approach of project methodology. A four-step approach was adopted for this proof of concept study to test whether it was possible to link Impact Case Studies (ICS) from the 2014 Research Excellence Framework (REF) exercise, to Researchfish Grant Agreements (GAs), and then to investigate the characteristics of the grants links to case studies compared to those that were not linked.

As illustrated in Figure 1, and described below, a four-step approach was adopted for this proof of concept study. In this paper, our goal was to enable manual time intensive tasks to be automated making a broader analysis of REF2014 more feasible. All linking was first attempted through semi-automated process, validated and when necessary supplemented by manual coding.

### Step 1: Linking ICS with Researchfish GA

**Figure 2.**
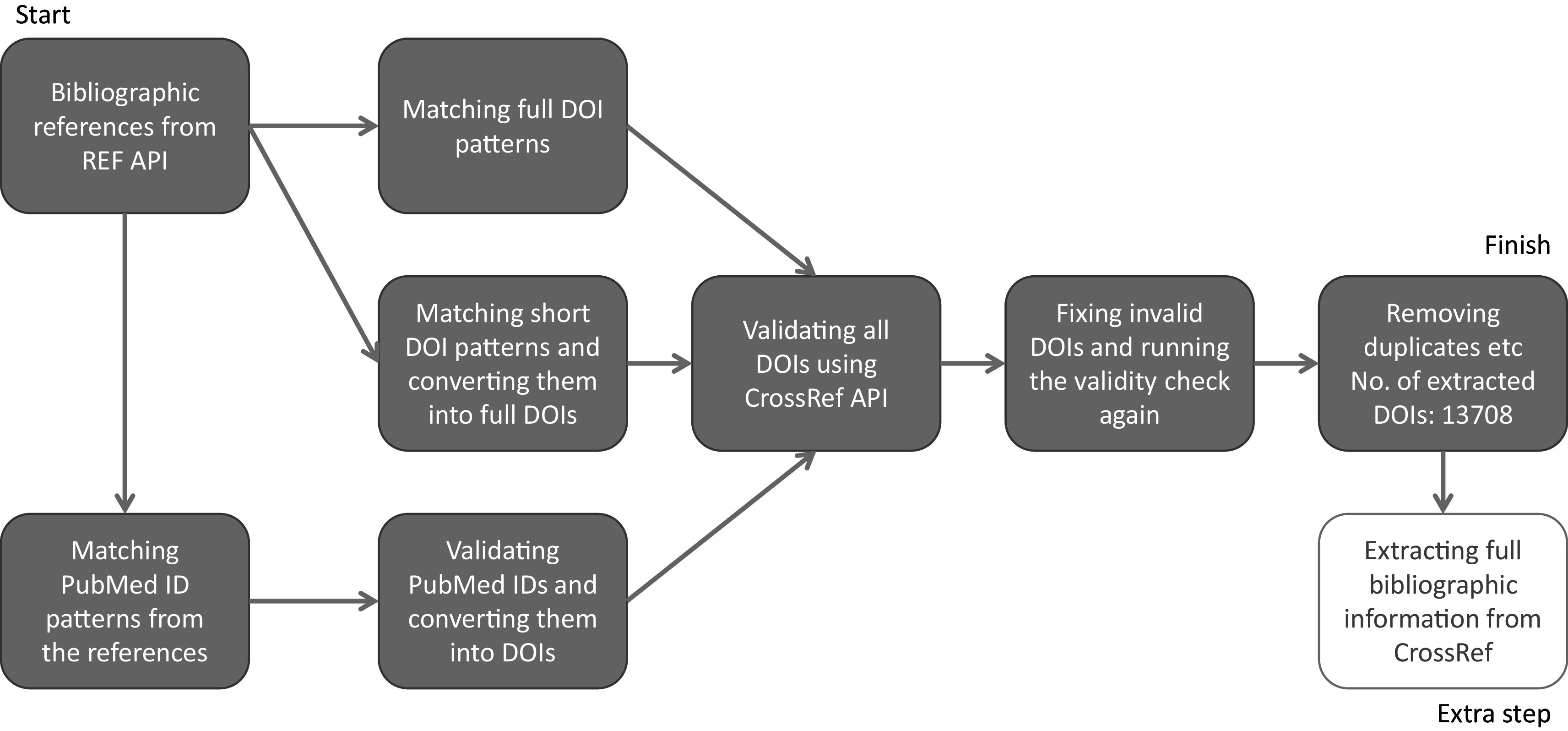
Process for cleaning and standardising digital object identifiers (DOIs) in Research Excellence Framework (REF) 2014 impact case studies (ICS). The process for matching bibliographic references from REF ICS needed to allow for variable types of persistent identifiers, namely DOIs and PubMed IDs, and then convert them all into valid DOIs for a consistent dataset to work on. This figure explains the process for cleaning, deduplicating and standardising the DOIs used for the study.

At the outset we tested whether it was possible to link REF ICS with Researchfish GAs using DOIs captured in both datasets. DOIs are persistent identifiers that remain fixed for the lifetime of a document and are widely used to identify academic, professional and government information such as journal articles and research reports. As such they occur in both REF ICS and Researchfish GAs providing a theoretical mechanism to link both datasets. However, linkage is complicated by varied and different approaches to indexing research publications. For example, in ICS researchers may use PubMed identifiers as well as both short and long forms of DOIs some may even provide no identifier at all. To take into account this variance a process was developed to clean and standardise DOIs to bibliographic information in the REF2014 ICS ( Figure 2).

### Step 2: Improving data linkage for a randomly selected group of 100 ICS

**Figure 3.**
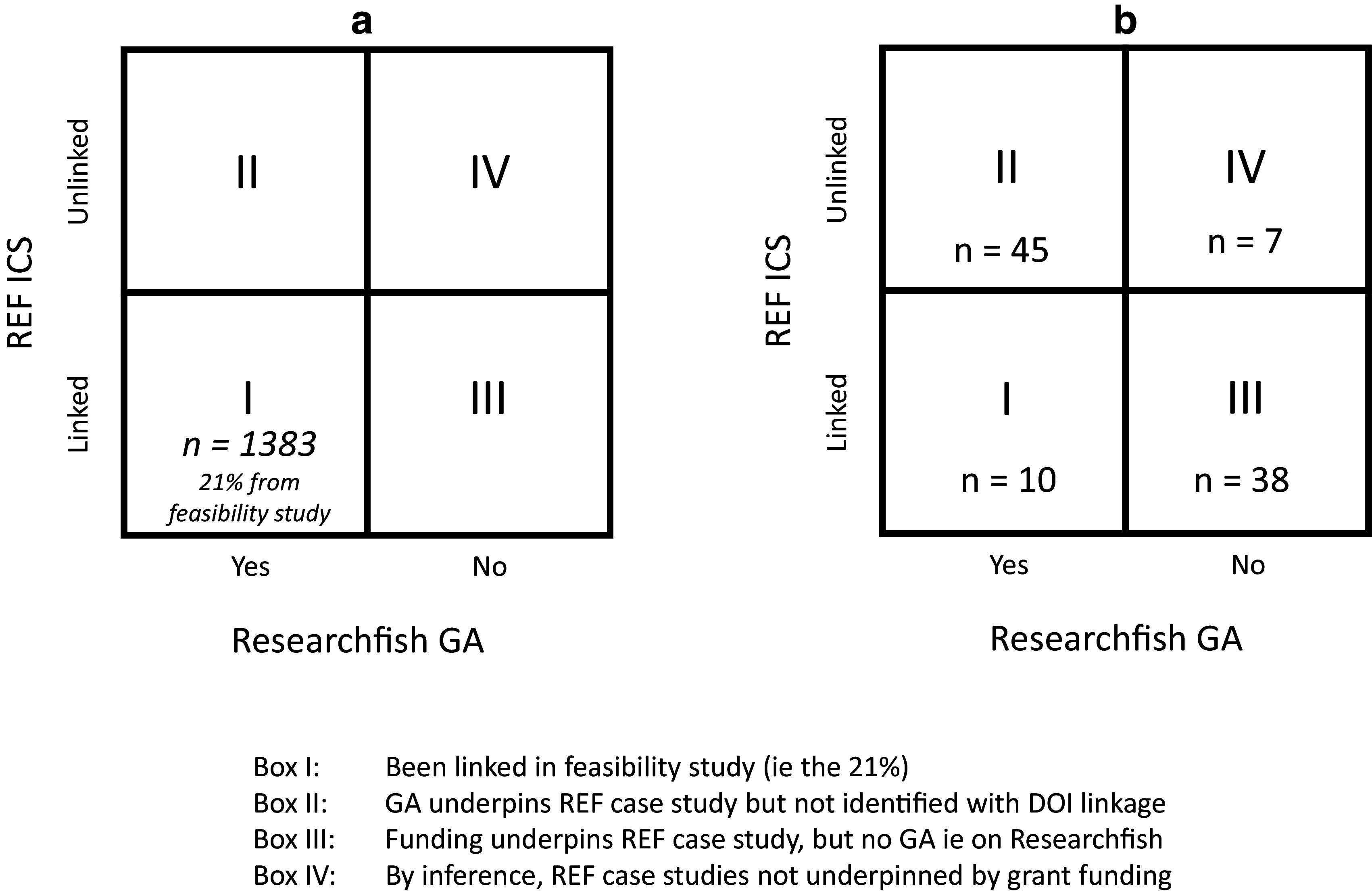
Conceptual overview for linking Research Excellence Framework (REF) 2014 impact case studies (ICS) with Researchfish grant agreements (GAs). This figure illustrates the next step of the process which aimed to assess the unlinked ICS (79%) taken from the Research REF 2014 dataset, and investigate whether they really did not have any underpinning research grant associated with them or are an ‘artefact’, either of (i) the process developed for Step 1 or (ii) have associated underpinning research grants that are not indexed on Researchfish. The box on the left represents the full set of case studies ( Figure 3a), and the different possibilities for each, and then the box on the right ( Figure 3b) represents 100 randomly selected case studies that could not be linked in step 1, and then results of further investigation on each. Box I: Linked in Step 1 (i.e. 21%). Box II: GA underpins REF case study but not identified with DOI linkage. Box III: Funding underpins REF case study, but no GA i.e. on Researchfish. Box IV: By inference, REF case studies not underpinned by grant funding.

A significant limitation of the first step was that only 21% of the ICS could be linked to GAs. The aim of step two was to assess, using the random number generator in Excel a sample of 100 case studies, whether the 79% of ‘unlinked’ case studies were ‘real’ i.e., do not have underpinning research grants associated with them or are an ‘artefact’, either of (i) the process developed for Step 1 or (ii) have associated underpinning research grants that are not indexed on Researchfish. This is illustrated in Figure 3a. On the horizontal axis is whether there is a Researchfish GA and on the vertical axis whether the ICS can be linked or not to the GA. The bottom left-hand box (I) indicates those 21% of ICS that could be linked to the GA in Step 1. The top left-hand box (II) are those GAs that do actually underpin an ICS but the semi-automated linkage process in Step 1 failed to make the match (that is they are an ‘artefact’ of the approach adopted). Similarly, the bottom right-hand box (III) have associated underpinning research grants but they are not indexed on Researchfish e.g. it may be an ICS that is underpinned by National Institutes of Health funding from the US or by a funder using Researchfish but they have chosen not to track that specific grant in the system for some reason. The final box (IV) in the top right- hand corner are those inferred ICS that have no underpinning research grants (whether indexed on Researchfish or from another non-indexed research funder).

The aim of this second step was in effect to populate this 2 × 2 matrix with 100 randomly selected case studies that could not be linked from Step 1. This involved developing and running other semi-automated searches to improve data matching and reading the case studies to identify additional information. Overall, four specific approaches were used. The first was enhanced DOI matching, which was effectively applying improvements to the initial approach applied in Step 1. The second approach involved extracting funding information from papers that were cited in the underpinning research on the ICS and then seeing whether that information could be matched to a GA. Typically, this involved a grant identifier in the paper and matching it with Researchfish. The third approach was using the structured funding information in the ICS and again seeing whether that could be matched to the Researchfish GA. The structured funding information included in the ICS database is limited to a small number (n = 16) of funders that were supported through the UK Science Budget disbursed by the Department of Business, Innovation & Skills (BIS) bodies and the Wellcome Trust (that co-funded the development of the ICS database). After this, qualitative judgement was used to compare, for example, the topic of the case study with titles and abstracts of GAs using keyword searches.

### Step 3: Additional qualitative analysis for unlinked ICS

The third step was based on qualitative analysis and involved reading the ICS to identify additional information to link to GA data and/or funding and once that was exhausted following up with telephone or email interviews with the authors of the remaining ICS to see whether the underpinning research was funded or not, and if so who funded it. Each of the ICS were read by three of the authors (DM, GR and JG) who met on a weekly basis to review their findings and ensure consistency in coding. The interviews were conducted by one author (GR).

### Step 4: Comparing the characteristics of linked ICS with GA with all GA

The final step involved comparing the linked ICS with GA to all GA, using a number of metrics derived from Researchfish output data. The purpose of this approach was to test whether such comparisons could be made and whether, in principle, they could provide interesting information for understanding the relationship between research funding and research impact. For this set we looked at both the original linked GA (i.e. the 21%) and those 55 ICS that we manage to link through the qualitative (Step 3) assessment.

## Results

**Figure 4.**
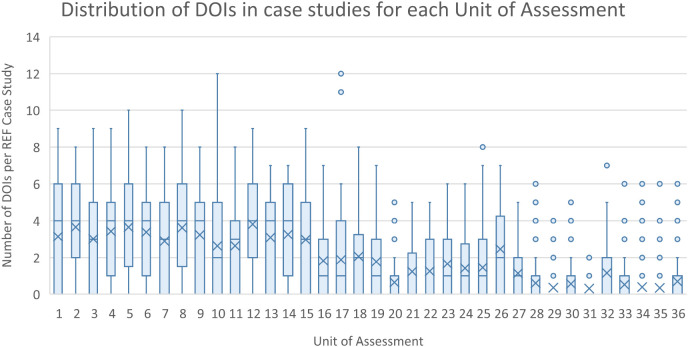
Distribution of digital object identifier (DOIs) in impact case studies (ICS) for each for each units of assessment (UoA). The figure shows the distribution of the number of extracted and validated publication DOIs for each of the ICS within each of the Research Excellence Framework UoA as a box plot.

**Figure 5.**
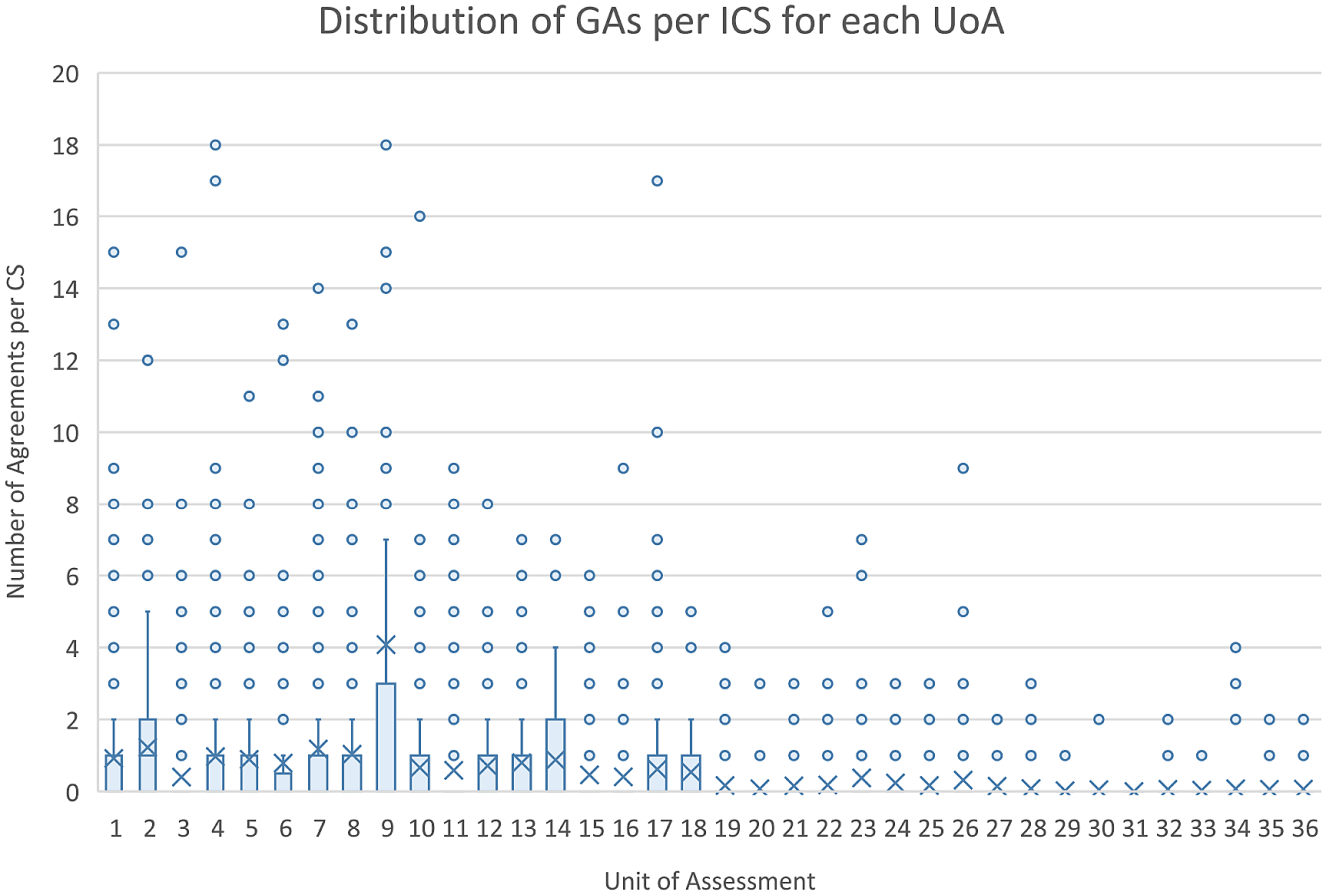
Distribution of grant agreements (GAs) per impact case studies (ICS) for each units of assessment (UoA). The figure shows the distribution of the number of GAs in Researchfish that were able to be linked to each of the ICS within each of the Research Excellence Framework UoA as a box plot.

The initial scraping of bibliographic information in the ICS (Step 1), resulted in 13,708 complete DOIs being identified. Of the 13,708 DOIs, 2805 (or 20%) could be matched to equivalent DOIs maintained in the Researchfish GA data. These GA DOIs are captured by a research object (i.e. a paper) either directly reported and attributed to a specific GA by the researchers (or their delegates) or automatically harvested based on funding acknowledgements in the papers themselves, and then subsequently confirmed by the researcher. This meant that 1383 of 6637 (i.e. 21%) non redacted case studies that can be downloaded from the REF impact case study database could be linked to specific research grants using this automated approach. As illustrated in Figures 4 and 5, the distribution of DOIs scrapped from ICS varied by the UOA, with greater numbers in Panels A and B than C and D, as was the number of linked GAs per ICS.

Table 1 summarises the results of Step 2 of developing semi-automated linkage by focusing on the randomly selected 100 case studies. As illustrated in this table the majority (57) of the ICS could be linked to GAs through these enhanced semi-automated approaches. The enhanced DOI matching included one ICS that would have been picked up in Step 1 due to an update in the data within Researchfish (the publication had been entered manually but a DOI for the publication was subsequently identified). DOIs for the remaining nine ICS were identified by extracting the bibliographic data from the case study, and using Crossref, to identify likely DOIs before validating and then discovering matches to GAs. Extracting the funding data from publications cited in the ICS and matching that to the GAs resulted in a further six linkages but the most significant addition was made through the use of the structured funding information captured in the ICS database, resulting in further 41 ICS being linked to GAs.

**Table 1.** Results of Phase 1, semi-automated linkage.

	Panel A (Life sciences)	Panel B (Engineering and Physical Sciences)	Panel C (Social Sciences)	Panel D (Arts and Humanities)	Total
Remaining cases	25	25	25	25	100
Enhanced DOI matching	23	22	22	23	90
Publication funding extraction	21	20	21	22	84
Structured funding information	12	9	12	10	43

The table shows the number of unmatched impact case studies (ICS) drawn from each of the four panels at each stage of the process. For example, in the case of Panel A the enhanced DOI (Digital Object Identifier) matching reduced the number of unmatched ICS in the sample from 25 to 23. This was further reduced to 21 unmatched case studies after using the publication funding extraction, and then finally reduced to 12 after extracting structured funding information.

The remaining 43 ICS were then read by three of the authors. This resulted in the identification of 34 ICS that had some form of underpinning research grant funding, but from a funder not indexed on Researchfish. For the remaining nine ICS, the authors were identified and contacted via email seeking information on any underpinning research funding and offering a response either by return via email or to arrange a telephone interview. Of the nine ICS, responses were received from six, and non-responses from three. Of the five ICS with additional information, two confirmed that they had some sort of research funding and were therefore allocated to Box III, with the remaining seven to Box IV of which five we confirmed no underpinning research funding.

As illustrated in Figure 3b, based on this analysis, the 2 × 2 matrix for the 100 randomly allocated case studies could be repopulated. This resulted in the majority of ICS (55 i.e. 10 Box I and 45 Box II) being linked to Researchfish GAs, and a further 38 having some form of underpinning research from funders not indexed on Researchfish. Only 7 of the 100 case studies seemed to have no identifiable external research grant funding associated with them and for three of this information was not able to be definitively confirmed.

Finally, and as illustrated in Table 2, the characteristics 1383 of 6637 (i.e. 21%) non redacted ICS and the additional 55 ICS that could be were subsequently linked through the more in-depth assessment were compared to the 82,603 GA in Researchfish (as of the 31/12/2013 i.e. at a similar time the ICS were submitted). Although these exploratory results should be treated with considerable caution they do through up a number of interesting observations. For example, it would seem that grant funding linked to REF impact case studies are more likely to: be longer in duration; be larger in value; have more publications; have policy influence appear sooner; have more collaborations; have higher levels of further funding; have more intellectual property. That said the discrepancy between the number of publications between the various columns does illustrate the risk of over interpreting these initial results.

**Table 2.** Exploratory differences between impact case study (ICS) with an underpinning grant agreement (GA) and all GAs.

Characteristics (Unit)	Updated ICS with underpinning GA identified in Steps 3-5 (n = 55)	All GA (n = 82603, as of 31/12/2013)	Original GA that were linked to ICS in Step 1 (n = 1383)
Median length of grant agreement (years)	3.7 years	3 years	3.5 years
Median value of grant agreement (£)	£270,345	£98,217	£320,132
Median number of publications	165	1	16
Median time for a policy influence	5 years	3 years	4 years
Median time for IP registration	4 years	3 years	3 years
Median number of collaborations	1	0	1
Median value of further funding	£125,000	£130,000	£170,000

The table shows the difference in characteristics between GAs in Researchfish depending on whether they were not linked to an ICS, were linked to an ICS as part of the original match, or were linked as part of the scoping study. The characteristics presented are: Median length of grant agreement (years), Median value of grant agreement (Great British Pounds £), Median number of publications, Median time for a policy influence, Median time for IP registration, Median number of collaborations, Median value of further funding (Great British Pounds £).

## Conclusions

The primary objective of this study was to test the feasibility and utility of linking REF ICS with Researchfish GAs to assess whether there is an opportunity to contribute to the broad literature on factors associated with research success. At its simplest, the answer is yes to both elements of this question. It proved feasible to link the two independent datasets and when linked generated interesting observations that *could* make an important contribution to the literature.

However, this conclusion should not be over interpreted as there are four of significant limitations with this proof of concept study. First, only a small proportion (21%) of the ICS could be liked using fully automated processes, however the more in-depth qualitative investigation showed that proportion could be significantly increased. Reassuringly, and as noted in Table 2, this increase did not alter the initial policy findings of the study i.e. there were differences between ICS that could be linked to GAs, vis-à-vis those that could not.

The second caveat is the data quality in both the ICS and the GAs, and particular the use of linkable identifiers such as DOIs. This issue may resolve itself as the proportion of publications reported within Researchfish that have DOIs has increased from *circa* 80% in 2006 to *circa* 92% in 2020. Similarly, the use of DOIs was automated in REF 2021 with case study authors having to confirm the details of underpinning research publications through third-party database when submitting ICS. This would suggest that in REF 2021 the number publications reported in ICS with DOIs will increase significantly (from around 26,000 reported by the 6,637 ICS in 2014).

The third caveat is that the Researchfish GA data is limited to those funders who use the platform (and which awards fit within the funders inclusion criteria for tracking in the Researchfish platform). Whilst this is the majority of UK funders it is notable in the list of non-indexed funders that there are a number of international funders who contributed the research that underpins ICS but the nature and characteristics of this research funding is excluded from the analysis. There is no a priori reason to think that their characteristics would necessarily be different to the funders indexed on Researchfish but that is an untested assumption that needs to be considered when interpreting the data from the two studies.

The final caveat is that we analysed the linkages between ICS and GA and we have yet to assess the size of those GAs, the number of GAs per case study or the nature of the GA funding beyond that presented in Table 2. All of this data is potentially available and something that could be examined in detail in a larger scaled up study, either of REF 2014 ICS or those from REF 2021.

The publication of the REF2021 ICS presents an opportunity to further develop this approach. Assuming a higher rate of automated linkage, say of around 60%, between the ICS and GAs (due to better use of DOIs) the application of the semi-automated and qualitative approaches developed here could be applied across the remaining *circa* 3000 case studies at not too great a cost. Back of the envelope calculation suggests that about 100 case studies could be processed a day. This means it would be practicable to scale up work presented in this paper, with the opportunity to make a significant contribution to our understanding of the characteristics of research funding underpinning societal impact.

## Data availability

### Source data

The REF 2014 data is publicly available for download, and is available for reuse as described by the REF 2014 data terms of use.

The publication information used was gathered from Crossref and PubMed.

Attribution information was used from Researchfish. This is not publicly available for reuse, but requests can be made to individual organisation listed at https://researchfish.com/the-members/. A large amount of the data collected via Researchfish and used in this study is publicly available for reuse via the Gateway to Research at https://gtr.ukri.org/.
