# Supplementary material for: Understanding the funding characteristics of research impact: A proof-of-concept study linking REF 2014 impact case studies with Researchfish grant agreements
Source: F1000Res. 2022 Sep 20;10:1291. Originally published 2021 Dec 17. [Version 3] doi: 10.12688/f1000research.74374.3 (PMC11109542; doi:10.12688/f1000research.74374.3)
Supplement: Peer review history

# Reviewer report: Reviewer response for version 3

**DOI:** 10.5256/f1000research.134255.r271191

**Published:** 2024-06-17

**Authors:** Maria Theresa Norn

**Affiliations:** 1 Aarhus University, Aarhus, Denmark

This proof of concept study proposes and describes an approach for linking REF impact studies with research grants reported to Research Fish. The approach is novel and well described.

 While the approach has several important limitations, these are clearly acknowledged in the paper. Overall, this appears to be a feasible and interesting approach worth further development and exploration, based on its potential to better link data on funding to data on research outputs and impact.

 Based on their application of this approach, the authors present some preliminary analysis of differences in grants that funded research reported in impact case studies vs. other research grant agreements. This was an interesting if very preliminary and explorative finding, which is but briefly touched upon in the paper. Given the exploratory nature of the paper, this is understandable, but I would very much encourage the authors to pursue the extended and scaled-up study that they themselves suggest in their concluding remarks in the article. In particular, I would hope to see the extended study dive deeper into the apparent characteristics of the funding of projects highlighted as REF impact case studies (can these findings be confirmed?) and reflect on the possible interpretations and implications of this application of their method, ideally by combining a scaled up version of the currently applied approach with qualitative data collected from the researchers behind the impact case studies. Also, I would like to see references to the literature on REF impact case studies: how well do the preliminary findings regarding the grants behind impact case studies align with existing studies of these cases? What do we know about the process and criteria for selection of these case studies, and to what extent may this explain the differences observed in the present paper? Ultimately, the interesting question is whether the type of approach presented in this paper can help us better understand how different characteristics of research funding may enable research with different impact characteristics, or whether it highlights which types of research projects are more likely to consider impact and to be selected as impact case studies (e.g. longer projects with larger budgets) - and what this ultimately tells us about the type of impact captured in these case studies.

 I would have liked to see some reflections on the potential applications of and insights from the method proposed in the paper, but given its methodological focus and the stated limitations, I can understand why this was not attempted. But, as mentioned, I encourage the authors to pursue the proposed scaled-up study.

Is the rationale for developing the new method (or application) clearly explained?

Yes

Is the description of the method technically sound?

Yes

Are the conclusions about the method and its performance adequately supported by the findings presented in the article?

Yes

If any results are presented, are all the source data underlying the results available to ensure full reproducibility?

Partly

Are sufficient details provided to allow replication of the method development and its use by others?

Yes

Reviewer Expertise:

Research impact assessment, research evaluation, studies of research funding, science policy.

I confirm that I have read this submission and believe that I have an appropriate level of expertise to confirm that it is of an acceptable scientific standard.

# Reviewer report: Reviewer response for version 3

**DOI:** 10.5256/f1000research.134255.r271194

**Published:** 2024-05-13

**Authors:** Mike Thelwall

**Affiliations:** 1 University of Sheffield, Sheffield, UK

How funding links to non-academic impacts is an important issue and this paper introduces a new way to help to assess this. The paper also mentions some characteristics of finding linked to REF impact case studies, showing that the method can give useful insights. The paper has a number of substantial limitations, which are mentioned appropriately in the paper.

Is the rationale for developing the new method (or application) clearly explained?

Yes

Is the description of the method technically sound?

Yes

Are the conclusions about the method and its performance adequately supported by the findings presented in the article?

Yes

If any results are presented, are all the source data underlying the results available to ensure full reproducibility?

Partly

Are sufficient details provided to allow replication of the method development and its use by others?

Yes

Reviewer Expertise:

Scientometrics

I confirm that I have read this submission and believe that I have an appropriate level of expertise to confirm that it is of an acceptable scientific standard.

# Reviewer report: Reviewer response for version 3

**DOI:** 10.5256/f1000research.134255.r271193

**Published:** 2024-05-09

**Authors:** Mark Reed

**Affiliations:** 1 Scotland’s Rural College, Edinburgh, UK

The introduction provides a clear rationale for the study, situating it in relevant literature to identify the gaps in knowledge that the paper addresses. This is probably not relevant enough to warrant a revision, but it may be worth noting that there have been previous studies analysing the relationship between funding sources and research impacts, focussing on conflicts of interests, where impacts were shown to align with the mission of the funder (particularly in biomedical sciences). If this is of interest and the authors can't find the sources, they can get in touch and I can try and dig these out. 

 The data sources are appropriate and I am pleased to see the REF impact data framed as a "proxy" for impact, though I wondered if it would be useful to more explicitly explain the biases inherent in this data source? 

 The methods are appropriate to answer the questions asked in this paper, and the automation provides a novel methodological contribution that could be used in future research. However, given the fact that manual analysis suggests that over half of the ICS not identified via automation were false negatives, and there was no manual check for false positives in the automated data set, I have questions about the wider applicability of this method.

 As such, I would interpret the findings differently to the more positive assertion given by the authors in the first paragraph of the conclusion. This is moderated by the second paragraph of the conclusion however, so it is perhaps justifiable. 

 The analysis is thorough and rigorous, including qualitative analysis and interviews with case study authors where necessary to collect the data needed for the analysis. 

 In the results section, it wasn't clear to me why the ICS identified automatically were presented separately to those that were identified manually via qualitative analysis. My understanding was that the sample of case studies that were manually coded had the purpose of testing the reliability of the automated procedure, to determine if those not identified automatically were false negatives (more than half were). Although the automated data set was not checked for false positives, I would have thought the two sources of linked ICS could be combined into a single data set, and wonder if it is worth presenting an additional column in Table 2 that integrates the two sources (alongside the two sources separately in the existing columns) as the primary source of findings for comparison with GAs not linked to ICS?

 Given the numbers involved, I would have thought that some descriptive statistics could have added value to the paper, making it possible to say with greater certainty whether or not there were "significant differences" between the number of publications, collaborators etc between GAs linked or not linked to ICS. If there are good reasons for not performing such tests (either parametric or non-parametric), perhaps the authors could provide these?

 I would be interested to see more of a research agenda in the conclusion around how causal links between the variables identified and impact might be explored. There may be many factors that could explain why larger, longer projects are more likely to be associated with impact without any causality necessarily being implied. 

 Minor point: In the last para of the results section, should "the characteristics 1383 of 6637" be "the characteristics of 1383 out of 6637"?

Is the rationale for developing the new method (or application) clearly explained?

Yes

Is the description of the method technically sound?

Yes

Are the conclusions about the method and its performance adequately supported by the findings presented in the article?

Yes

If any results are presented, are all the source data underlying the results available to ensure full reproducibility?

Yes

Are sufficient details provided to allow replication of the method development and its use by others?

Yes

Reviewer Expertise:

Research impact, engagement and participation, including:Understanding knowledge and impact (co-)production processesBoundary organisations and knowledge brokerageManaging conflict in the natural environmentStakeholder analysisParticipatory scenario developmentParticipatory monitoring and evaluationParticipatory modellingDeliberative processes and techniquesShared values for the natural environment

I confirm that I have read this submission and believe that I have an appropriate level of expertise to confirm that it is of an acceptable scientific standard, however I have significant reservations, as outlined above.

# Reviewer report: Reviewer response for version 3

**DOI:** 10.5256/f1000research.134255.r138739

**Published:** 2022-10-26

**Authors:** Daniele Rotolo

**Affiliations:** 1 SPRU (Science Policy Research Unit), University of Sussex, Brighton, UK

I am happy with the revisions the authors have submitted.

Is the rationale for developing the new method (or application) clearly explained?

Yes

Is the description of the method technically sound?

Yes

Are the conclusions about the method and its performance adequately supported by the findings presented in the article?

Yes

If any results are presented, are all the source data underlying the results available to ensure full reproducibility?

Partly

Are sufficient details provided to allow replication of the method development and its use by others?

Partly

Reviewer Expertise:

science policy and innovation studies

I confirm that I have read this submission and believe that I have an appropriate level of expertise to confirm that it is of an acceptable scientific standard.

# Reviewer report: Reviewer response for version 2

**DOI:** 10.5256/f1000research.133836.r138052

**Published:** 2022-05-19

**Authors:** Adam Kamenetzky

**Affiliations:** 1 National Institute for Health Research Central Commissioning Facility, Twickenham, UK

**Editorial Note from F1000Research – 19th May 2022:**

***Please note, in accordance with the advice of the submitting reviewer, we have changed three erroneous references to ‘CrossRef’ to read as ‘EuropePMC’. The mentions of ‘CrossRef’ were made accidentally; no substantial changes to the body, style or intention behind the review have been made.***

 I thank the authors for their replies and for their efforts to submit a second version of this article, which addresses a majority of my comments. Nonetheless I remain concerned with, and have discussed with the journal’s editorial team, the lack of underpinning data being made available for a research article of this nature.

 While I acknowledge that this study is a proof-of-concept, it nonetheless sets a precedent. Publication of underpinning data - and as an absolute minimum, the subset of these data that already exist in (and/or are derived from) data already in the public domain - seems necessary to meet the Journal’s data availability policies, ensure appropriate transparency and reproducibility, and would greatly enhance the potential for this work to inform further analyses. Additionally, as a point of principle, I believe that there is a strong public interest case in making publicly available both the data and the results of analyses derived from any publicly-listed linkages between public-/charity-funded research grant awards (which presumably make up a majority of UK-based funder awards reporting via Researchfish), resultant research outputs, and publicly-available research impact case studies.

 I hope that in considering and addressing these further comments the authors are able to maximise the potential for individuals and research organisations to make use of and derive further value from these data, and future impact assessment efforts of this kind.

**Comment #1**

 For the purposes of transparency and so that readers are aware of the general availability of Researchfish data, it would be helpful for the authors to clarify and add wording in the introduction to the effect that output data entered by researchers linked to grant awards, and any funder-specific analyses of these data, are held by Researchfish behind a paywall and provided to funders and research organisations under a commercial licence. The relationship between Researchfish and its parent company, Interfolio, should also be made explicit. Those authors whose affiliations are listed as “Interfolio UK” should declare their interests accordingly. 

**Comment #2**

 I would encourage the authors to speak with the journal’s editorial team to determine how they might comply to the fullest extent possible with its policies for data accessibility and to discuss an appropriate course of action to address any data protection issues, as necessary. Based on my understanding of Researchfish and publication indexing systems such as EuropePMC, I provide the following specific recommendations that I would be grateful if the authors might consider and respond to, as part of any further dialogue.

 For any data already in the public domain, and/or analyses derived from data in the public domain, these data should be published. My understanding - and I would be grateful if the authors might clarify this - is that whenever a researcher attributes an indexed publication to a grant award within Researchfish, this information is pushed to EuropePMC, which publicly lists both the funder’s name and an award reference alongside the indexed publication. In these cases, where award-publication linkages are already in the public domain, I cannot see how publication of this linkage data would be in breach of any terms of collection or data regulations? For the subset of 100 randomly-selected impact case studies, the authors state that they obtained further information via emails and interviews with researchers based on award information which was already in the public domain. And surely all linkages of publications to REF impact case studies are *de facto* in the public domain, given the public availability of the REF2014 impact case study database and underpinning research?

 For any data underpinning this analysis that are not in the public domain (e.g. award-publication linkages that are not listed on EuropePMC, and/or analyses of wider non-publication outputs held by Researchfish), sufficient descriptive information (e.g. aggregated total numbers of grant awards matched and/or other outputs, by funder) should be published to allow the reader to specifically request such data from the relevant funding organisations. In this case, as a minimum, the authors may wish to consider presenting aggregate data in a similar fashion to the recent pre-publication by Ohid Yaqub and colleagues (available at https://doi.org/10.31235/osf.io/qw873) which also explored linkages between UKRI research application pathways to impact statements, and REF2014 impact case studies.

Is the rationale for developing the new method (or application) clearly explained?

Partly

Is the description of the method technically sound?

Yes

Are the conclusions about the method and its performance adequately supported by the findings presented in the article?

Yes

If any results are presented, are all the source data underlying the results available to ensure full reproducibility?

No

Are sufficient details provided to allow replication of the method development and its use by others?

Partly

Reviewer Expertise:

Research impact assessment, research evaluation, research on research, studies of research funding organisations, science policy research & analysis.

I confirm that I have read this submission and believe that I have an appropriate level of expertise to confirm that it is of an acceptable scientific standard, however I have significant reservations, as outlined above.

# Response

**Published:** 2022-09-15

**Authors:** Beverley Sherbon

**Affiliations:** Interfolio UK, UK

Reviewers comments: I thank the authors for their replies and for their efforts to submit a second version of this article, which addresses a majority of my comments. Nonetheless I remain concerned with, and have discussed with the journal’s editorial team, the lack of underpinning data being made available for a research article of this nature.

 While I acknowledge that this study is a proof-of-concept, it nonetheless sets a precedent. Publication of underpinning data - and as an absolute minimum, the subset of these data that already exist in (and/or are derived from) data already in the public domain - seems necessary to meet the Journal’s data availability policies, ensure appropriate transparency and reproducibility, and would greatly enhance the potential for this work to inform further analyses. Additionally, as a point of principle, I believe that there is a strong public interest case in making publicly available both the data and the results of analyses derived from any publicly-listed linkages between public-/charity-funded research grant awards (which presumably make up a majority of UK-based funder awards reporting via Researchfish), resultant research outputs, and publicly-available research impact case studies.

 I hope that in considering and addressing these further comments the authors are able to maximise the potential for individuals and research organisations to make use of and derive further value from these data, and future impact assessment efforts of this kind.

**Reviewer Comment #1**

 For the purposes of transparency and so that readers are aware of the general availability of Researchfish data, it would be helpful for the authors to clarify and add wording in the introduction to the effect that output data entered by researchers linked to grant awards, and any funder-specific analyses of these data, are held by Researchfish behind a paywall and provided to funders and research organisations under a commercial licence. The relationship between Researchfish and its parent company, Interfolio, should also be made explicit. Those authors whose affiliations are listed as “Interfolio UK” should declare their interests accordingly. 

**Authors Response #1** 

 The data referenced in this proof of concept study is not held by Researchfish behind a paywall and then provided to funders and research organisations under a commercial licence. Funders subscribe to use Researchfish to collect information on the outputs, outcomes, and impacts of their funded research. This data is requested by the funders, and belongs to the funders.

 Regarding the 'relationship between Researchfish and its parent company', Researchfish is not a company. The authors are affiliated with Interfolio UK, which is the name of the company that manages the Researchfish application. To avoid any possibility of further misunderstanding the authors have updated the conflict of interest statement to make this clearer. Please also note that between versions 2 and 3 of this paper Interfolio UK was acquired by Elsevier (June 2022), part of RELX.

**Reviewer Comment #2**

 I would encourage the authors to speak with the journal’s editorial team to determine how they might comply to the fullest extent possible with its policies for data accessibility and to discuss an appropriate course of action to address any data protection issues, as necessary. Based on my understanding of Researchfish and publication indexing systems such as EuropePMC, I provide the following specific recommendations that I would be grateful if the authors might consider and respond to, as part of any further dialogue.

 For any data already in the public domain, and/or analyses derived from data in the public domain, these data should be published. My understanding - and I would be grateful if the authors might clarify this - is that whenever a researcher attributes an indexed publication to a grant award within Researchfish, this information is pushed to EuropePMC, which publicly lists both the funder’s name and an award reference alongside the indexed publication. In these cases, where award-publication linkages are already in the public domain, I cannot see how publication of this linkage data would be in breach of any terms of collection or data regulations? For the subset of 100 randomly-selected impact case studies, the authors state that they obtained further information via emails and interviews with researchers based on award information which was already in the public domain. And surely all linkages of publications to REF impact case studies are *de facto* in the public domain, given the public availability of the REF2014 impact case study database and underpinning research?

 For any data underpinning this analysis that are not in the public domain (e.g. award-publication linkages that are not listed on EuropePMC, and/or analyses of wider non-publication outputs held by Researchfish), sufficient descriptive information (e.g. aggregated total numbers of grant awards matched and/or other outputs, by funder) should be published to allow the reader to specifically request such data from the relevant funding organisations. In this case, as a minimum, the authors may wish to consider presenting aggregate data in a similar fashion to the recent pre-publication by Ohid Yaqub and colleagues (available at https://doi.org/10.31235/osf.io/qw873) which also explored linkages between UKRI research application pathways to impact statements, and REF2014 impact case studies.

**Authors Response #2**

 The authors of the publication contacted the journal’s editorial team before submission to determine whether publication would be possible given the nature of the data involved and the journal’s policies. There are permissions for publication – grant linkage information collected through Researchfish to be made available to EuropePMC, who make this information publicly available alongside the other data they hold on publication – grant linkages. The authors link to this, and other publicly available data used and provide information on how to contact funders and request access to the funders’ data that is not otherwise public. The authors do not have the permissions needed to provide access to the funders’ data in the manner suggested. The purpose of this publication is not to sell a proprietary dataset, or to create a table of organisations based around their connections to impact case studies, but to explain how the authors found that the connections between funding and REF 2014 case studies are far greater than an initial analysis of the REF data would otherwise suggest. The authors describe the approach taken, the methods applied, and the data used. Anyone who wishes to do so could reproduce this study and come to the same conclusion.

# Reviewer report: Reviewer response for version 1

**DOI:** 10.5256/f1000research.78121.r116005

**Published:** 2022-02-02

**Authors:** Daniele Rotolo

**Affiliations:** 1 SPRU (Science Policy Research Unit), University of Sussex, Brighton, UK

Many thanks for the opportunity to read this interesting proof of concept. The challenges of generating data that integrate funding and research output (including impact) remains relatively unaddressed despite the importance of such type of data source to inform policymaking. Hence, this paper provides an interesting and promising contribution in this direction. The main argument of the paper is also clear to follow. I have provided below some suggestions that I hope are helpful to strengthen some aspects of the paper.

 First, depending on what could be disclosed, the paper would benefit of a more detailed description of ResearchFish GA data and of their coverage. This would allow the reader to reach a better understanding of what could explain the “missing links” (e.g. in terms of which UK and non-UK funders are not included in the data).

 Second, the analysis is focussed on how many ICSs could be linked to ResearchFish GAs. However, it is unclear whether the matching was also assessed in terms of proportion of ResearchFish GAs that could be linked in the case of ICSs with multiple funding sources. How does the matching perform in these cases?

 Finally, as you also argued, the results reported in Table 2 should be cautiously interpreted (I suggest adding the word “exploratory” in the caption of the table). This is particularly true since, as discussed above, an ICS could be supported by more than one funding within and/or outside ResearchFish GA data. This seems an important point to clarify in the paper.

 Minor comments:

 - Figure 1 would be easier to read if the four circles were aligned in sequence from left to right (similar to Figure 2).

 - Panel A and B in Figure 3 could be combined.

 Thanks again for the opportunity to read your paper.

Is the rationale for developing the new method (or application) clearly explained?

Yes

Is the description of the method technically sound?

Yes

Are the conclusions about the method and its performance adequately supported by the findings presented in the article?

Yes

If any results are presented, are all the source data underlying the results available to ensure full reproducibility?

Partly

Are sufficient details provided to allow replication of the method development and its use by others?

Partly

Reviewer Expertise:

innovation studies, emerging technologies, scientometrics, corporate science

I confirm that I have read this submission and believe that I have an appropriate level of expertise to confirm that it is of an acceptable scientific standard, however I have significant reservations, as outlined above.

# Response

**Published:** 2022-05-06

**Authors:** Beverley Sherbon

**Affiliations:** Interfolio UK, UK

**Comment 1:**

 Many thanks for the opportunity to read this interesting proof of concept. The challenges of generating data that integrate funding and research output (including impact) remains relatively unaddressed despite the importance of such type of data source to inform policymaking. Hence, this paper provides an interesting and promising contribution in this direction. The main argument of the paper is also clear to follow. I have provided below some suggestions that I hope are helpful to strengthen some aspects of the paper.

 First, depending on what could be disclosed, the paper would benefit of a more detailed description of ResearchFish GA data and of their coverage. This would allow the reader to reach a better understanding of what could explain the “missing links” (e.g. in terms of which UK and non-UK funders are not included in the data).

**Response to comment 1:**

 A paragraph has been added describing the coverage of Researchfish data. 

 Currently Researchfish has data on over 195,000 Grant Agreements, with over 80% of them from the UK.  These UK data report on 268,000 different outputs, outcomes or impact before December 31 ^st^ 2013 (the cut off period for REF 2014).  All the major funders in the UK (i.e. UKRI, Wellcome Trust and other medical research charities) use Researchfish and over the period 2006-2013 this accounted for between £2.5-£4.0 billion of research funding each year.  It, should, however be noted that Researchfish does not cover research that is funded by other means, for example block grants to universities (QR funding), direct donation from philanthropists and other self initiated research. 

**Comment 2:**

 Second, the analysis is focussed on how many ICSs could be linked to ResearchFish GAs. However, it is unclear whether the matching was also assessed in terms of proportion of ResearchFish GAs that could be linked in the case of ICSs with multiple funding sources. How does the matching perform in these cases?

**Response to comment 2:**

 This is a really good question but one we consider to be out of scope of the current paper.  We are currently working on a project that tries to estimate what proportion of grant funding leads to some form of impact (of which being linked to an ICS could be a proxy indicator).  The literature on this topic is very sparse and thus we do belief it is a fruitful area to explore but, as noted, is probably beyond the remit of the current work.

**Comment 3:**

 Finally, as you also argued, the results reported in Table 2 should be cautiously interpreted (I suggest adding the word “exploratory” in the caption of the table). This is particularly true since, as discussed above, an ICS could be supported by more than one funding within and/or outside ResearchFish GA data. This seems an important point to clarify in the paper.

**Response to comment 3:**

 This change has been made

**Comment 4:**

 Minor comments:

 - Figure 1 would be easier to read if the four circles were aligned in sequence from left to right (similar to Figure 2).

 - Panel A and B in Figure 3 could be combined.

**Response to comment 4:**

 Both figures have been slightly altered in the next version of the paper. Another reviewer commented on our language of Panel A and Panel B in Figure 3 runs the risk of being confused with the REF panels, so we have renamed these.

# Reviewer report: Reviewer response for version 1

**DOI:** 10.5256/f1000research.78121.r115998

**Published:** 2022-01-11

**Authors:** Adam Kamenetzky

**Affiliations:** 1 National Institute for Health Research Central Commissioning Facility, Twickenham, UK

The authors present a novel and timely proof-of-concept study to investigate linkages between research funding and the reporting of subsequent research impact narratives, using two large data sources - Researchfish (an online platform for reporting research outcomes to funders), and the Research Excellence Framework (a national research impact assessment exercise in the UK) 2014 impact case study database.

 The introduction traces a brief history of studies of research impact and in doing so provides a helpful context to the study.

**Comment #1:** *[Intro, para 3] *The sentence beginning *“As described below…”* and the remainder of this paragraph starts to describe the study methods and (final sentence) goes on to note *“interesting observations…”* and study limitations. Rather than noting these here partially, I would suggest these aspects might be more clearly and fully described in later sections of the article? Instead, it may be helpful for the authors to describe here in a sentence or two their specific rationale for setting out to link research funding and research impact data (e.g. reflections on why and for whom this particular analysis might be useful).

 The authors clearly describe the two data sources that form the basis for the study. 

 The article methods and results describe and present insights from a four-step process undertaken by the authors to link Researchfish Grant Awards (GA) with REF impact case studies (ICS), primarily using commonly-reported publications and associated funding information as the locus for further analysis. Overall, I found these sections to be detailed and well described. However I do have a couple of queries relating to the availability of source data and software used for the analysis, and would recommend that the authors consult relevant sections of the journal’s data guidelines (noted with reference to specific sub-sections in comments #2 & #5 below) to ensure that the journal’s open data policy is being met to the fullest extent possible. 

**Comment #2:** *[Methods, para 1] *The authors refer both to “the code” and (subsequently) “the [linkage] algorithm”, but use these terms somewhat interchangeably and do not provide details. I would suggest that the authors a) clearly describe the software used for analysis (see data guidelines, section 1.1), and b) make available the source code/algorithm that they developed, as part of the study data (see data guidelines, section 2.1.2) or alternatively provide a rationale for why this cannot be shared as part of the data availability statement (see data guidelines, section 2.3).

**Comment #3:** *[Methods, figure 3] *It may be helpful for the authors to use alternative terms than “Panel A” / “Panel B” for the two boxes presented in this figure, to avoid any confusion with REF review panels. Also, both here and at other points in the article the authors refer to a “feasibility study” and “Step 1” somewhat interchangeably, when describing the initial semi-automated step to link REF ICS and Researchfish GA via commonly-reported publications; it would be helpful to be consistent when describing these as “Step 1”, throughout. Lastly, both in the left-hand box and at various points in the main body of the article the authors refer to “[the] 21%”; it would be helpful to clarify here the absolute numbers of ICS / GA that were linked / unlinked in each quadrant (as per the right-hand box), as well as the total numbers of ICS being analysed (as I understand it, n=6,637 at step 1, and n=100 at step 2?)

**Comment #4:** *[Methods, Step 3]* The authors describe a process to interview (via telephone or email) original authors of the REF ICS, to ascertain whether underpinning research was funded, and who funded it. While appreciably much of this information may already be in the public domain (though not necessarily referred to explicitly in REF ICS), this process could be considered primary data collection for the purposes of the research study. Please could the authors either a) provide information around ethical considerations and approvals (which would ordinarily include informed consent processes undertaken with interviewees upfront, provision of copies of interview protocols that were used, etc.) for the purposes of this study, or b) if ethical approvals were not sought, consider this data as appropriate for scoping purposes only and not appropriate for inclusion as research data as part of the qualitative analysis undertaken in Step 3? If the latter, it may be helpful for the authors to reflect on the scalability of the method outlined in this study and the relative benefits of this additional interview step (e.g. in terms of the number of additional matches it provided) versus the additional requirements (e.g. in seeking appropriate ethical approvals, informed consent, opportunity costs for interviewers/interviewees etc.), were this analysis to be carried out across a larger sample of ICS?

**Comment #5:** *[Results, para 1] *The authors report a total of 1,383 research grants as successfully matched to impact case studies via automated methods. These linkages would seem novel and the underlying data of value to a range of audiences (not least the research funding organisations themselves). Similarly to comment #2, above, and in line with the journal’s data guidelines, I would suggest that the authors consider publishing these data, at least to include a set of searchable GA-DOI-ICS matches? Alternatively, it would be helpful to understand which specific elements of these data are considered unsuitable or unavailable to publish, if they are not already in the public domain (e.g. as the authors note via the UKRI Gateway to Research, or via other funders’ open publication of grant award data). As appreciably this may not be trivial given the number of funders whose grantees’ data are held in Researchfish, the authors might give an indication of any data protection issues that could arise from any potential extension or scaling up of this method, and in seeking to publish - as would seem appropriate - the underlying matched (e.g. GA-DOI-ICS) data.

**Comment #6:** *[Results, Table 2 & final para] *The authors refer to a “scoping study” however I am a bit unclear as to the nature or sequencing of this in relation to the 4-step process previously described in the methods and results. Additionally, the median number of publications associated with impact case studies in this category (= *“Updated ICS…”*) noted as 165, would seem rather discrepant with the equivalent figure for Step 1 (= *“Original GA…”*) noted as 16. Perhaps both these aspects could be clarified and/or explained?

 The authors conclude that the method is both feasible and useful as a means to link two independent datasets with information on the progress of research towards wider societal benefits, and I would agree that there is broad value in efforts to explore such linkages further. In particular, as outlined above, I would recommend to the authors that to the greatest degree possible, such data are made publicly available to encourage further analysis and ensure reproducibility of results. With the REF2021 exercise mandating the use of unique publication IDs (via DOI), funder IDs (via GRID) and grant award reference numbers, this kind of linkage analysis ought to become increasingly possible using publicly-available data. The authors’ efforts to show proof of concept in this regard are thus particularly timely.

 I thank the authors for the opportunity to review this study and would be happy to review any revised version or findings from any extension of the method, as appropriate.

Is the rationale for developing the new method (or application) clearly explained?

Partly

Is the description of the method technically sound?

Yes

Are the conclusions about the method and its performance adequately supported by the findings presented in the article?

Yes

If any results are presented, are all the source data underlying the results available to ensure full reproducibility?

No

Are sufficient details provided to allow replication of the method development and its use by others?

Partly

Reviewer Expertise:

Research impact assessment, research evaluation, research on research, studies of research funding organisations, science policy research & analysis.

I confirm that I have read this submission and believe that I have an appropriate level of expertise to confirm that it is of an acceptable scientific standard, however I have significant reservations, as outlined above.

# Response

**Published:** 2022-05-06

**Authors:** Beverley Sherbon

**Affiliations:** Interfolio UK, UK

Thank you for reviewing our paper and the considered comments.

**Comment #1:** *[Intro, para 3] *The sentence beginning *“As described below…”* and the remainder of this paragraph starts to describe the study methods and (final sentence) goes on to note *“interesting observations…”* and study limitations. Rather than noting these here partially, I would suggest these aspects might be more clearly and fully described in later sections of the article? Instead, it may be helpful for the authors to describe here in a sentence or two their specific rationale for setting out to link research funding and research impact data (e.g. reflections on why and for whom this particular analysis might be useful).

**Response to comment 1:**

 We have reflected on this comment but have decided against making any changes. We think this section provides a useful route map for the reader in navigating the rest of the paper. We have, however, noted one type which we have corrected in this paragraph (changing ‘ ..study while an eye …’ to ‘ … study with an eye …’)

**Comment #2:** *[Methods, para 1] *The authors refer both to “the code” and (subsequently) “the [linkage] algorithm”, but use these terms somewhat interchangeably and do not provide details. I would suggest that the authors a) clearly describe the software used for analysis (see data guidelines, section 1.1), and b) make available the source code/algorithm that they developed, as part of the study data (see data guidelines, section 2.1.2) or alternatively provide a rationale for why this cannot be shared as part of the data availability statement (see data guidelines, section 2.3).

**Response to comment 2:**

 We agree with the author that there is some confusion in the language and we have therefore made changes throughout the manuscript avoid the specific and perhaps misleading use of ‘code’ and ‘algorithm’. In the study we were basically running iteratively different searchers terms and processes and in the text we have clarified that.

**Comment #3:** *[Methods, figure 3] *It may be helpful for the authors to use alternative terms than “Panel A” / “Panel B” for the two boxes presented in this figure, to avoid any confusion with REF review panels. Also, both here and at other points in the article the authors refer to a “feasibility study” and “Step 1” somewhat interchangeably, when describing the initial semi-automated step to link REF ICS and Researchfish GA via commonly-reported publications; it would be helpful to be consistent when describing these as “Step 1”, throughout. Lastly, both in the left-hand box and at various points in the main body of the article the authors refer to “[the] 21%”; it would be helpful to clarify here the absolute numbers of ICS / GA that were linked / unlinked in each quadrant (as per the right-hand box), as well as the total numbers of ICS being analysed (as I understand it, n=6,637 at step 1, and n=100 at step 2?)

**Response to comment 3:**

 We have changed ‘Panel A’ to Figure 3a and ‘Panel B’ to Figure 3b in throughout. We have also added ‘n=1383’ in Figure 3a for the ICS that could be linked to Researchfish GA. Note the numbers in the other boxes are unknown, hence Step 2 in the study. Finally, we have amended the text to talk solely about Step 1 (as opposed to feasibility study) as suggested.

**Comment #4:** *[Methods, Step 3]* The authors describe a process to interview (via telephone or email) original authors of the REF ICS, to ascertain whether underpinning research was funded, and who funded it. While appreciably much of this information may already be in the public domain (though not necessarily referred to explicitly in REF ICS), this process could be considered primary data collection for the purposes of the research study. Please could the authors either a) provide information around ethical considerations and approvals (which would ordinarily include informed consent processes undertaken with interviewees upfront, provision of copies of interview protocols that were used, etc.) for the purposes of this study, or b) if ethical approvals were not sought, consider this data as appropriate for scoping purposes only and not appropriate for inclusion as research data as part of the qualitative analysis undertaken in Step 3? If the latter, it may be helpful for the authors to reflect on the scalability of the method outlined in this study and the relative benefits of this additional interview step (e.g. in terms of the number of additional matches it provided) versus the additional requirements (e.g. in seeking appropriate ethical approvals, informed consent, opportunity costs for interviewers/interviewees etc.), were this analysis to be carried out across a larger sample of ICS?

**Response to comment 4:**

 Given the emergent nature of this proof-of-concept study ethics approval was not sought for the email interviews. It should be noted that the information used to approach the nine interviewees was all in the public domain. The template email that we sent interviewees is available from the authors. The main idea of this proof-of-concept study was to see whether such data linkage could occur within the need to approach individuals. The findings here, as discussed in the conclusion, suggest this may be the case with improved DOIs linkage in more contemporary Researchfish GA data and the REF 2021 case studies. In other words, it is not envisaged in a future study that interviews will be needed and thus the last point is redundant.

**Comment #5:** *[Results, para 1] *The authors report a total of 1,383 research grants as successfully matched to impact case studies via automated methods. These linkages would seem novel and the underlying data of value to a range of audiences (not least the research funding organisations themselves). Similarly to comment #2, above, and in line with the journal’s data guidelines, I would suggest that the authors consider publishing these data, at least to include a set of searchable GA-DOI-ICS matches? Alternatively, it would be helpful to understand which specific elements of these data are considered unsuitable or unavailable to publish, if they are not already in the public domain (e.g. as the authors note via the UKRI Gateway to Research, or via other funders’ open publication of grant award data). As appreciably this may not be trivial given the number of funders whose grantees’ data are held in Researchfish, the authors might give an indication of any data protection issues that could arise from any potential extension or scaling up of this method, and in seeking to publish - as would seem appropriate - the underlying matched (e.g. GA-DOI-ICS) data.

**Response to comment 5:**

 We have added some data on Researchfish, but for the reasons acknowledged have developed this point.

**Comment #6:** *[Results, Table 2 & final para] *The authors refer to a “scoping study” however I am a bit unclear as to the nature or sequencing of this in relation to the 4-step process previously described in the methods and results. Additionally, the median number of publications associated with impact case studies in this category (= *“Updated ICS…”*) noted as 165, would seem rather discrepant with the equivalent figure for Step 1 (= *“Original GA…”*) noted as 16. Perhaps both these aspects could be clarified and/or explained?

**Response to comment 6:**

 We have updated Table 2 to refer to 'Steps 2-4’ instead of ‘scoping study’ and corrected the n to 55 (an error in the original draft). The text already notes the caution needed in interpreting the table but we have amplified that with an additional sentence.

**Comment #7:**

 In particular, as outlined above, I would recommend to the authors that to the greatest degree possible, such data are made publicly available to encourage further analysis and ensure reproducibility of results.

**Response to comment 7:**

 The authors of the paper fully agree with the reviewer, and data is available wherever possible in line with the necessary terms of collection and data regulations.
